# Developmental competence and antigen switch frequency can be uncoupled in *Trypanosoma brucei*

**DOI:** 10.1073/pnas.1912711116

**Published:** 2019-10-21

**Authors:** Kirsty R. McWilliam, Alasdair Ivens, Liam J. Morrison, Monica R. Mugnier, Keith R. Matthews

**Affiliations:** ^a^Institute for Immunology and Infection Research, School of Biological Sciences, University of Edinburgh, EH9 3FL Edinburgh, Scotland, United Kingdom;; ^b^Department of Veterinary Sciences, Experimental Parasitology, Ludwig Maximilians Universität München, 80752 Munich, Germany;; ^c^Biomedical Center Munich, Department of Physiological Chemistry, Ludwig Maximilians Universität München, 82152 Planegg-Martinsried, Germany;; ^d^Roslin Institute, Royal (Dick) School of Veterinary Studies, University of Edinburgh, EH25 9RG Midlothian, Scotland, United Kingdom;; ^e^Johns Hopkins Bloomberg School of Public Health, Johns Hopkins University, Baltimore, MD 21205

**Keywords:** *Trypanosoma*, antigenic variation, differentiation, immune evasion, parasite

## Abstract

African trypanosomes use an extreme form of antigenic variation to evade host immunity, involving the switching of expressed variant surface glycoproteins by a stochastic and parasite-intrinsic process. Parasite development in the mammalian host is another feature of the infection dynamic, with trypanosomes undergoing quorum sensing (QS)-dependent differentiation between proliferative slender forms and arrested, transmissible, stumpy forms. Longstanding experimental studies have suggested that the frequency of antigenic variation and transmissibility may be linked, antigen switching being higher in developmentally competent, fly-transmissible, parasites (“pleomorphs”) than in serially passaged “monomorphic” lines that cannot transmit through flies. Here, we have directly tested this tenet of the infection dynamic by using 2 experimental systems to reduce pleomorphism. Firstly, lines were generated that inducibly lose developmental capacity through RNAi-mediated silencing of the QS signaling machinery (“inducible monomorphs”). Secondly, de novo lines were derived that have lost the capacity for stumpy formation by serial passage (“selected monomorphs”) and analyzed for their antigenic variation in comparison to isogenic preselected populations. Analysis of both inducible and selected monomorphs has established that antigen switch frequency and developmental capacity are independently selected traits. This generates the potential for diverse infection dynamics in different parasite populations where the rate of antigenic switching and transmission competence are uncoupled. Further, this may support the evolution, maintenance, and spread of important trypanosome variants such as *Trypanosoma brucei evansi* that exploit mechanical transmission.

## Significance

Trypanosomes are blood-borne protozoa that cause human sleeping sickness and the livestock disease, nagana. These extracellular parasites sustain infection through their ability to change their surface proteins, a process called antigenic variation. They also promote their own transmission by development in the bloodstream to so-called “stumpy forms” generated in response to parasite density. Earlier studies have proposed that reduced capacity for stumpy formation also generates reduced antigen switch frequency, suggesting these processes are mechanistically coupled. Here, by silencing the density-sensing pathway or selecting parasites less able to generate stumpy forms, we demonstrate that these processes are under independent selection. This has relevance for parasite virulence, spread, and competition and for the selection of trypanosome species directly transmitted by biting flies.

Trypanosomes are blood- and tissue-dwelling parasites that persist extracellularly through their capacity for antigenic variation, allowing them to sustain prolonged infections despite their exposure to host immunoglobulins (1). Antigenic variation results from the expression on the parasite surface of a dense coat of variant surface glycoproteins (VSGs) that shield invariant proteins from immune recognition. The parasite genome encodes ∼2,500 *VSG* genes, mainly located in subtelomeric regions and held as a silent archive (2, 3). For expression, *VSG* genes are transcribed from 1 of ∼15 telomeric expression sites (ESs), of which only 1 is active at a time. This monoallelic expression is maintained through association with a subnuclear expression site body (4) as well as the stoichiometry of the ES-associated factor, VEX1 (5). Antigen switching can occur by changing the active expression site or by gene conversion of a *VSG* into the active expression site, either as intact genes from a silent location, or through the assembly of chimeras. The latter is necessitated because many *VSG* genes in the silent archive are interrupted by stop codons and frameshifts, such that productive antigenic variation requires mosaic VSGs to be generated by gene conversion from several incomplete donors (6). Deep sequencing approaches analyzing early and chronic infections have established that many antigen types can comprise part of each parasitemic wave although early parasitemias can be dominated by one or a few types (7, 8).

A further component that shapes the infection dynamic is the parasite differentiation from proliferative slender forms to nonproliferative, transmissible stumpy forms (9). Slender forms replicate as the parasitemia is established but with increasing parasite numbers, a density-sensing phenomenon induces the differentiation to stumpy forms. This quorum sensing (QS)-type process is induced by oligopeptide signals (10) and transduced via a signaling pathway that involves protein kinases and phosphatases as well as gene expression regulators and hypothetical proteins of unknown function (11). The generation of stumpy forms assists spread of the parasite because these forms preferentially survive uptake by tsetse flies, the vector for most African trypanosome species.

With long-term serial passage between rodent hosts or in culture, trypanosomes lose the capacity to generate stumpy forms and become “monomorphic” (12, 13). Because these cells do not undergo growth arrest in response to parasite density, they are highly virulent. These laboratory-adapted lines are also reported to be more antigenically stable than transmissible “pleomorphic” trypanosomes capable of full development through tsetse flies (14). Estimates of antigen switch frequency in laboratory-adapted trypanosomes differ depending on the experimental method used but are reported to be relatively low—typically 1 × 10^−6^ switches/cell/generation (15, 16). In contrast, recently fly transmitted trypanosomes exhibit much higher switch frequencies, around 1 × 10^−3^ switches/cell/generation (17). This has led to the dogma that developmental capacity and antigenic variation are coupled processes during the laboratory adaptation of trypanosome lines, with pleomorphic cells able to switch at high frequency while monomorphic cells switch at low frequency.

## Results

**Fig. 1.**
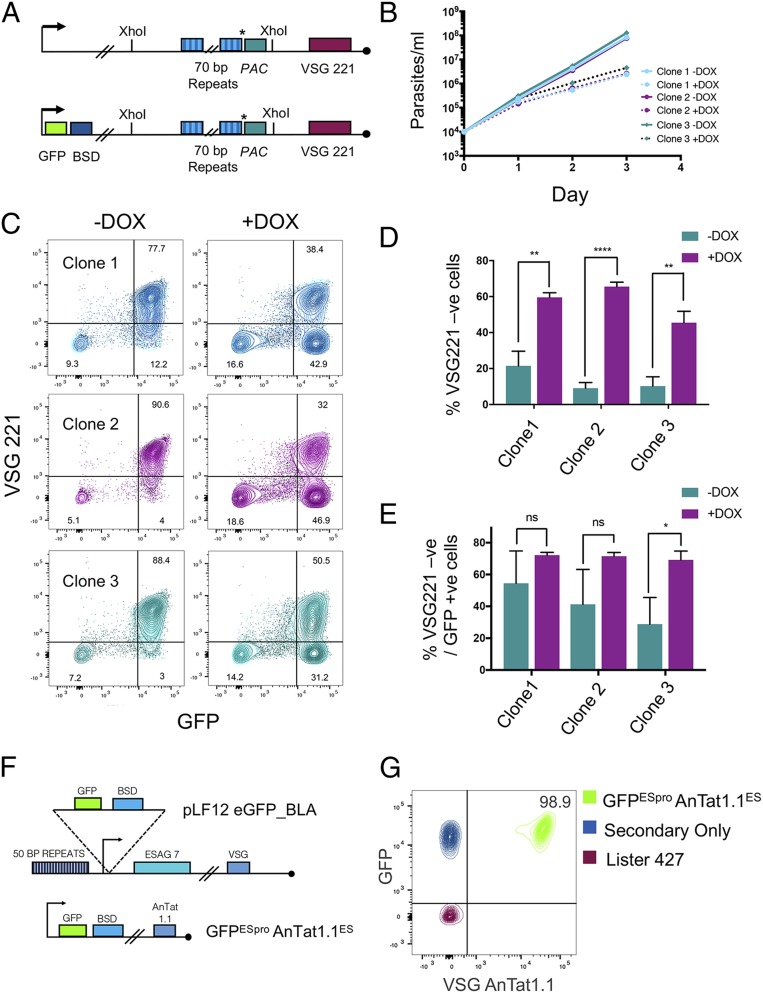
Monitoring VSG switches using flow cytometry. (*A*) Reporter constructs used to assess the FACS-based switch assay in Lister 427 cells. (*Upper*) The VSG 221 ES of the VSG^up^ strain contained an I-*Sce*I recognition sequence (*) and the *PAC* gene adjacent to the 70-bp repeats. Tetracycline-inducible I-*Sce*I was encoded from a rRNA spacer locus. (*Lower*) Incorporation of GFP into the VSG221 ES permits expression site switching to be monitored; a VSG221-specific antibody detects VSG221 expression. (*B*) Cumulative in vitro growth of 3 uninduced (bold line) and induced (dotted line) VSG^up^ pLF12 I-*Sce*I eGFP_*BLA* clones. Three replicates were performed for each condition and clone. Doxycycline was added on day 0 and replaced following addition of fresh HMI-9. Data represent the mean ± SD, *n* = 3. (*C*) VSG 221 and GFP expression of cells isolated on day 3 post I*Sce*-I induction. A total of 10,000 events were captured for each replicate. The plots show the data for all 3 triplicates superimposed. The mean proportions of cells in the quadrants are shown (*n* = 3). VSG 221 expression was determined by α-VSG 221 staining; 221 ES activity was determined with GFP positivity. (*D*) The percentage of cells classified as VSG 221 negative on day 3 after induction of I-*Sce*I expression. Data represent the mean ± SD (*n* = 3). **P ≤ 0.01, ****P ≤ 0.0001, Student’s *t* test. (*E*) The percentage of VSG 221 negative cells that were GFP positive on day 3. Data represent the mean ± SD (*n* = 3). *P ≤ 0.05, Student’s *t* test. ns, not significant, *P* > 0.05. (*F*) GFP^ESpro^AnTat1.1^ES^ *HYP2*, GFP^ESpro^AnTat1.1^ES^ *NEK*, and GFP^ESpro^AnTat1.1^ES^ *DYRK* RNAi cell lines express GFP through incorporation of pLF12 eGFP_*BLA* into the VSG AnTat1.1 ES promoter region. (*G*) FACS analysis of 10,000 cells demonstrated that the GFP^ESpro^AnTat1.1^ES^ RNAi reporter cell lines were ∼99% VSG AnTat1.1 and GFP positive. VSG AnTat1.1 positivity was measured by α-VSG AnTat1.1 binding and GFP positivity by fluorescence emission in the FITC channel alone. Cells without secondary antibody and Lister 427 cells acted as negative controls.

To monitor antigen switch frequency in parasites that were competent or not for differentiation, we established a fluorescence-activated cell sorting (FACS)-based assay able to detect antigen switches and capable of distinguishing the mechanism used to achieve switching. This entailed targeting a GFP reporter construct proximal to the VSG expression site promoter region and monitoring both GFP fluorescence and VSG labeling. With the exception of unlikely recombination events between the closely adjacent (462 bp) promoter and fluorescent reporter, this assay discriminates switches generated by recombination within the 40- to 60-kb expression site (GFP^+^/VSG^−^) from expression site switches (GFP^−^/VSG^−^). Initially, we validated the VSG switch assay by incorporating the GFP reporter construct into Lister 427 monomorphic cells capable of doxycycline-controlled expression of the I-*Sce*I nuclease, which promotes VSG switches by cleavage at a site in the VSG 221 ES (BES1), immediately adjacent to the 70-bp repeats (18) (Fig. 1*A*). Upon I-*Sce*I induction and the subsequent generation of a double strand break within the active ES, cell growth was inhibited (Fig. 1*B*) as previously reported (18). Further, FACS-based analysis using a VSG 221-specific antibody and GFP fluorescence confirmed that the assay detected a switch away from VSG 221 expression that was predominantly a consequence of recombination within the expression site (Fig. 1 *C–E*).

The same approach was then applied to *Trypanosoma brucei* EATRO 1125 pleomorphic cell lines expressing VSG AnTat1.1 and engineered to individually silence the expression of 3 genes required for QS-based stumpy formation (11), thereby testing whether antigen switch frequency changes with the loss of developmental competence. The GFP open reading frame (ORF) was integrated just downstream of the AnTat1.1 ES promoter (Fig. 1*F* and *SI Appendix*, Fig. S1) (19) in individual cell lines capable of tetracycline-inducible RNAi-mediated gene silencing of *HYP2* (Tb927.9.4080), *NEK17* (hereafter referred to as “*NEK*”; Tb927.10.5930 to 5950, these genes being cross-targeted by RNAi) and *DYRK* (Tb927.10.15020), thus generating GFP^ESpro^AnTat1.1^ES^ *HYP2*, GFP^ESpro^AnTat1.1^ES^ *NEK*, and GFP^ESpro^AnTat1.1^ES^ *DYRK* RNAi cell lines. In parental cells of *T. brucei* EATRO 1125 transfected with the GFP reporter construct, 98.9% of cells were GFP^+^/VSG AnTat1.1^+^ (detected using an antibody specific for VSG AnTat1.1), whereas control parasites (*T. brucei* Lister 427) without the reporter construct and expressing a distinct VSG (VSG221) were negative for both GFP and VSG AnTat1.1 (Fig. 1*G*).

**Fig. 2.**
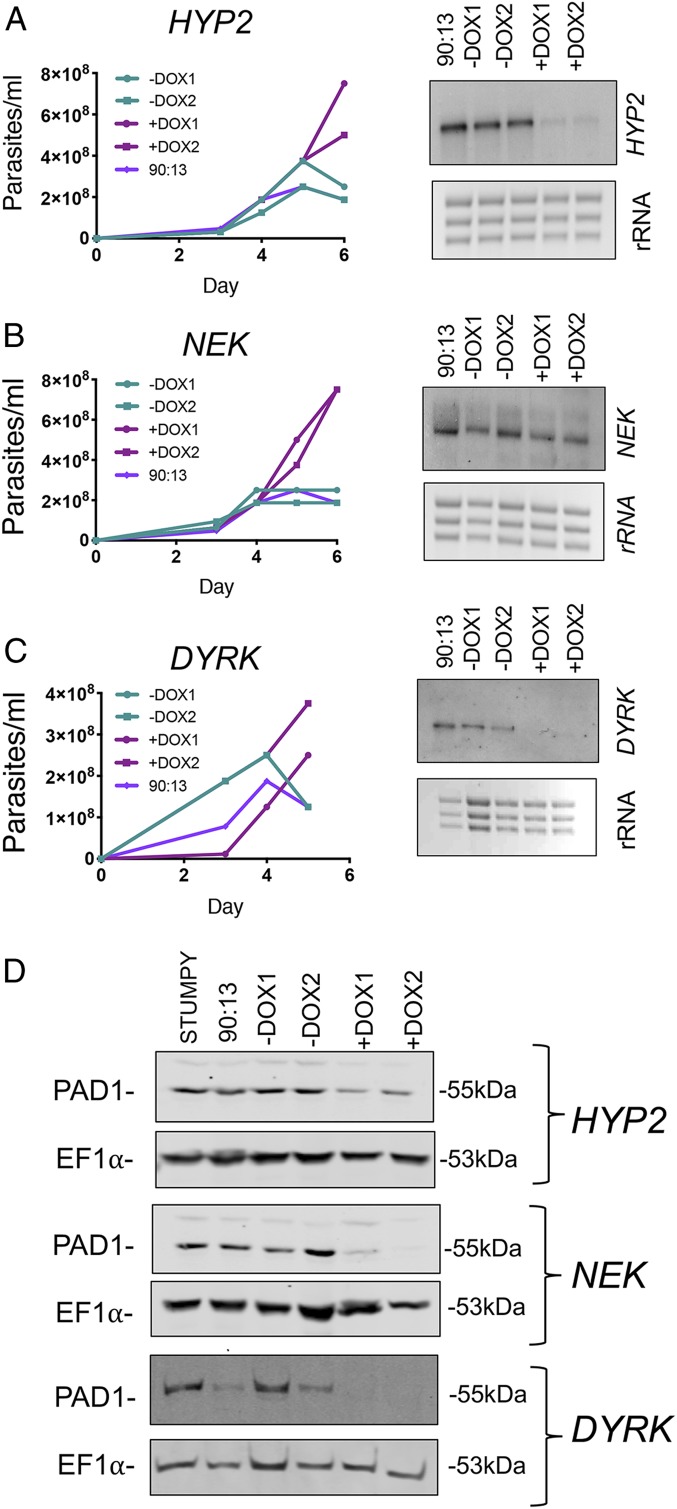
Silencing QS components in GFP^ESpro^AnTat1.1^ES^ reporter lines generates inducible monomorphism. (*A*–*C*, *Left*) In vivo growth of the GFP^ESpro^AnTat1.1^ES^ *HYP2*, GFP^ESpro^AnTat1.1^ES^ *NEK*, and GFP^ESpro^AnTat1.1^ES^ *DYRK* RNAi cell lines. Induction of RNAi generated populations of cells that grew to high parasitemia and were slender in morphology, contrasting with uninduced stumpy forms. Northern blot detection (*A*–*C*, *Right*) of *HYP2*, *NEK*, and *DYRK* transcripts revealed varying degrees of gene knockdown upon induction with doxycycline. AnTat1.1 90:13 parental cells were used as a control. (*D*) Western blot detection of PAD1 protein; AnTat1.1 90:13 parental cells which had arrested as stumpy forms were used as a control; EF1α provides a loading control.

Having generated RNAi lines for QS signaling components containing the reporter construct, we confirmed that these exhibited inducible loss of developmental competence. The GFP-ES tagged *HYP2*, *NEK*, and *DYRK* RNAi lines were grown in mouse infections, with gene silencing induced or not, by provision of doxycycline to the rodent drinking water (Fig. 2 *A–C*). In each case the capacity for stumpy formation was reduced (Fig. 2 *A–C*, *Left*), and the transcript for each target was inducibly depleted (Fig. 2 *A–C*, *Right*). The parasites proliferated to high parasitemia when induced and these parasites retained a slender morphology (*SI Appendix*, Fig. S2 *A*–*C*). Further, the induced parasites also exhibited lower expression of the stumpy form-specific marker, PAD1 (20) (Fig. 2*D*). Hence the cells showed reduced stumpy formation upon RNAi activation, a phenomenon we term “inducible monomorphism.”

The antigen switch frequency of each of the inducible monomorph lines was then determined in vitro. Specifically, parasites were grown in culture prior to induction and then cloned by serial dilution. Clonal populations were then expanded into increasing culture volumes to a total cell number of ∼2 × 10^7^, ensuring that cells were maintained in logarithmic growth (*SI Appendix*, Fig. S3). This prevented stumpy formation in the uninduced cells and ensured approximately the same number of replications in the uninduced and induced populations. In triplicated analyses, the uninduced populations of GFP^ESpro^AnTat1.1^ES^ *HYP2*, GFP^ESpro^AnTat1.1^ES^ *NEK*, and GFP^ESpro^AnTat1.1^ES^ *DYRK* RNAi cells took 10 d (range = 3, *n* = 18), encompassing 25.84 population doublings (±0.5, *n* = 18) to generate at least 2 × 10^7^ cells, whereas the induced cells also took 10 d, encompassing 25.69 population doublings (±0.81, *n* = 17). Induced and uninduced cell populations for each RNAi line were then analyzed by FACS to measure the switch frequency in the populations and the contribution of expression site switching and DNA recombination in any of the VSG AnTat1.1 negative cells (these being GFP^−^/VSG AnTat1.1^−^ or GFP^+^/VSG AnTat1.1^−^, respectively).

**Fig. 3.**
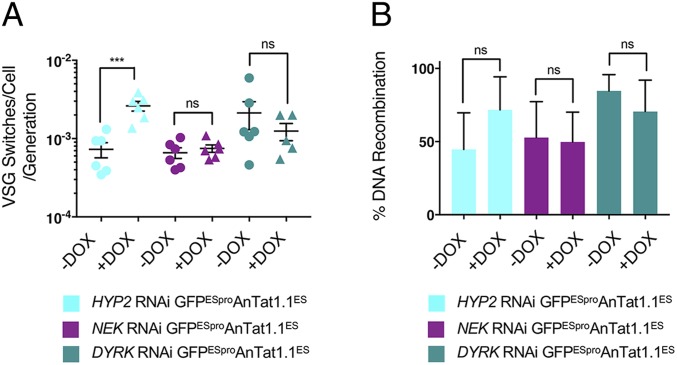
Reducing developmental competence by RNAi does not affect VSG switch frequency. (*A*) VSG switches/cell/generation for the uninduced and induced GFP^ESpro^AnTat1.1^ES^ *HYP2* (blue), GFP^ESpro^AnTat1.1^ES^ *NEK* (plum), and GFP^ESpro^AnTat1.1^ES^ *DYRK* (teal) RNAi lines. There was no significant difference in VSG switches/cell/generation upon the induction of *NEK* or *DYRK* RNAi (*P* > 0.05, Student’s *t* test). VSG switching was significantly increased upon knockdown of *HYP2* (***P ≤ 0.001, Student’s *t* test). Data represent the mean ± SD (*n* = 6, except for the induced *DYRK* RNAi population where *n* = 5). (*B*) The percentage of switched GFP^ESpro^AnTat1.1^ES^ *HYP2* (blue), GFP^ESpro^AnTat1.1^ES^ *NEK* (plum), and GFP^ESpro^AnTat1.1^ES^ *DYRK* (teal) RNAi cells which had switched their expressed VSG by DNA recombination (i.e., VSG AnTat1.1^−^/GFP^+^). Both *DYRK* populations and the induced *HYP2* population switched preferentially by DNA recombination, whereas the VSG switches detected in both *NEK* populations and the uninduced *HYP2* population were evenly split between ES and DNA recombination switches. There was no significant difference in VSG switch mechanism between the pleomorphs and inducible monomorphs for each cell line (*P* > 0.05, Student’s *t* test). ns, not significant, *P* > 0.05.

When the frequency of antigenic variation for the uninduced GFP^ESpro^AnTat1.1^ES^ *HYP2*, GFP^ESpro^AnTat1.1^ES^ *NEK*, and GFP^ESpro^AnTat1.1^ES^ *DYRK* RNAi lines was determined, switch rates of 7.27 × 10^−4^ ± 0.0004, 6.6 × 10^−4^ ± 0.0003, and 2.13 × 10^−3^ ± 0.002 switches/cell/generation, respectively, were observed (Fig. 3*A*). These rates were similar to the reported switch frequency of fly-transmitted parasites (17). However, in each of the 3 induced populations exhibiting relative monomorphism, the respective VSG switch rates did not drop to the estimated 10^−7^ to 10^−5^ VSG switches/cell/generation of laboratory-adapted trypanosomes. In fact, there was an increase in switch frequency upon induction of RNAi against *HYP2* (to 2.61 × 10^−3^ ± 0.0009 switches/cell/generation *P* = 0.0008, Student’s *t* test). *NEK* knockdown caused a slight increase in VSG switch rate and *DYRK* knockdown decreased the VSG switch rate; however, neither of these changes was significant (*P* > 0.05, Student’s *t* test) (Fig. 3*A*). In addition to the overall frequency of antigenic variation, we analyzed the mechanism of switching. The VSG AnTat1.1 negative cells in the uninduced GFP^ESpro^AnTat1.1^ES^ *DYRK* RNAi populations were mostly GFP positive (reflecting a DNA recombination-based switch), while the VSG AnTat1.1 negative cells in the uninduced GFP^ESpro^AnTat1.1^ES^ *HYP2* and GFP^ESpro^AnTat1.1^ES^ *NEK* RNAi populations were equally distributed between the GFP positive and negative gates. The analysis revealed that there was no significant difference (*P* > 0.05, Student’s *t* test) in the relative frequency of DNA recombination-mediated switches with respect to in situ switches in the uninduced or induced populations for each RNAi cell line, although these data showed considerable variability (Fig. 3*B*).

**Fig. 4.**
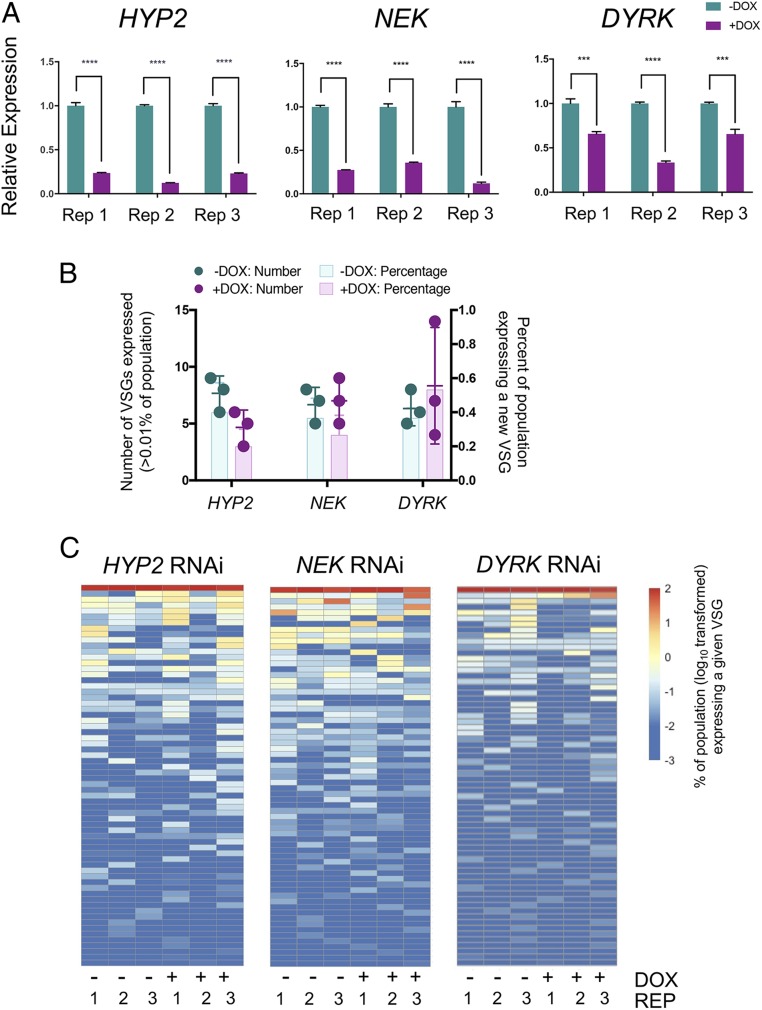
Reducing developmental competence by RNAi does not affect VSG expression diversity. (*A*) Validation of *HYP2* (*Left*), *NEK* (*Middle*), and *DYRK* (*Right*) transcript knockdown upon RNAi induction. Uninduced replicates are shaded in teal, induced are in plum. cDNA was generated from 300 ng pre-MACS RNA. Data represent the mean ± SD (*n* = 3). ***P ≤ 0.001, ****P ≤ 0.0001. (*B*) The number of VSGs (dot symbols) expressed by over 0.01% of the total population was counted for the uninduced (−DOX, teal) and induced (+DOX, plum) GFP^ESpro^AnTat1.1^ES^ *HYP2*, GFP^ESpro^AnTat1.1^ES^ *NEK*, and GFP^ESpro^AnTat1.1^ES^ *DYRK* RNAi lines. The percentage of cells in the populations that consisted of non-VSG AnTat1.1 expressors is represented by the colored bars. Data represent the mean ± SD (*n* = 3). (*C*) VSG expression heatmap showing the percentage (log_10_ transformed) of each replicate of the uninduced and induced *HYP2*, *NEK*, and *DYRK* RNAi populations expressing a given VSG. The percentage of the population expressing a given VSG was calculated by the VSG-specific RPKM divided by the total population RPKM. Each row represents a different VSG. The color intensity scale on the *Right* ranges from dark red (100% of the population) to dark blue (0.001% of the population). The identity of the VSG represented by each row of a heatmap is consistent between the uninduced and induced samples of a given GFP^ESpro^AnTat1.1 RNAi cell line, but not between the heatmaps of the 3 GFP^ESpro^AnTat1.1 lines themselves (except for VSG AnTat1.1, which is always represented by the *Top* row). “−“ and “+” represent the absence or presence of doxycycline.

To explore whether the diversity in the generated expressed VSGs was different in the induced and uninduced populations we exploited VSGseq (7) before and after enrichment for VSG AnTat1.1 negative cells by magnetic-activated cell sorting (MACS). Each “inducibly monomorphic” line (*HYP2*, *NEK*, and *DYRK*) was grown from clones to a total of at least 1.25 × 10^8^ cells ± RNAi induction (*SI Appendix*, Fig. S4) providing sufficient material to analyze the switch events by FACS, cell cycle status by fluorescence microscopy, and to generate cDNA after MACS enrichment (increasing depth of analysis in the populations). To generate the required cell number following cloning, populations were grown for ∼12 d, and the derived material was analyzed to confirm that the proportion of proliferative cells was not significantly different between the induced and uninduced samples (*P* > 0.05, Student’s *t* test). This ensured that the analyses of switch frequency were not confounded by cell differentiation to arrested stumpy forms. The depletion of the target transcript by RNAi in the induced samples was also validated, demonstrating a mean 80.3% depletion of *HYP2* transcript in the induced samples, and 75% depletion of *NEK*. For *DYRK*, the efficiency of depletion was lower, with 55% of transcript remaining in the induced populations (Fig. 4*A*).

Quantitatively, VSGseq analysis showed that there was no change in the overall number of VSG types detectable at >0.01% of reads expressed in the unsorted induced cell lines (Fig. 4*B*). Overall, in the MACS sorted populations, expression of 106 individual VSGs was detected. However, there was no consistent pattern of VSG expression either within the triplicates, or within the pleomorphic and inducibly monomorphic populations themselves. No VSGs were consistently expressed at levels >10-fold greater in the inducibly monomorphic populations compared to the pleomorphic populations and, furthermore, no VSG was ever found in all 3 replicates of either the uninduced or induced population and not observed in the corresponding uninduced or induced populations (Fig. 4*C*). Mosaic VSGs were not detected in any of the VSG sets analyzed using a <98% identity threshold. Thus, the inducible monomorphism generated by the gene silencing of 3 components of the QS signaling pathway did not generate a change in the frequency or diversity of expressed VSG in the populations.

**Fig. 5.**
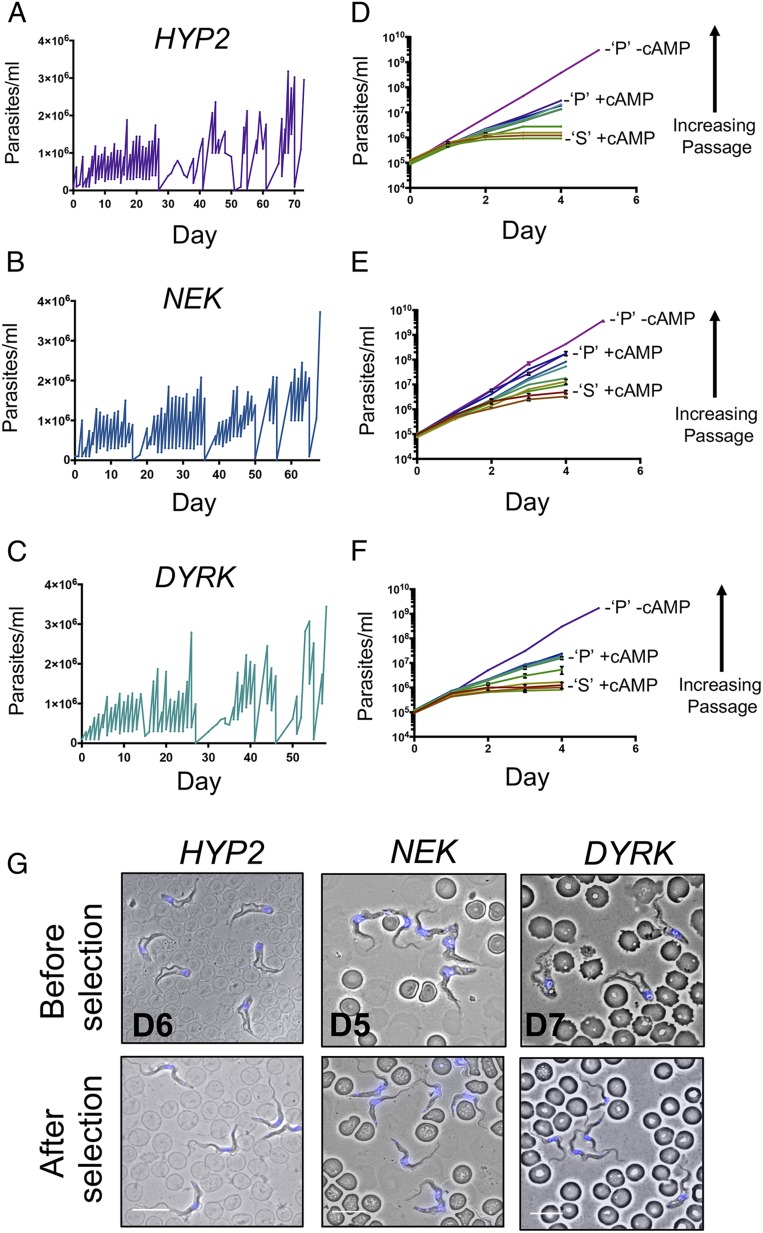
Selection of monomorphism by culture passage. (*A*–*C*) In vitro selection for monomorphism by repeated passage at high parasite density. Growth profiles for the uninduced GFP^ESpro^AnTat1.1^ES^ *HYP2* (*A*; selected for 73 d), GFP^ESpro^AnTat1.1^ES^ *NEK* (*B*; selected for 68 d), and GFP^ESpro^AnTat1.1^ES^ *DYRK* (*C*; selected for 58 d) RNAi cell lines. (*D*–*F*) In vitro cumulative growth curves of the GFP^ESpro^AnTat1.1^ES^ *HYP2* (*D*), GFP^ESpro^AnTat1.1^ES^ *NEK* (*E*), and GFP^ESpro^AnTat1.1^ES^ *DYRK* (*F*) RNAi populations in response to 8pCPT-cAMP exposure with increasing passage time. The cumulative growth of the respective starting (“S”) pleomorphic populations in the presence of 8pCPT-cAMP and the respective final passaged (“P”) populations in the absence of 8pCPT-cAMP are shown for reference. (*G*) Morphological analysis of cells isolated on the final day of infection (day 5 to day 7, depending on the cell line). Parental cells became morphologically stumpy (*Upper*), while selected cells (*Lower*) remained largely long and slender. Nuclear and kinetoplast DNA was visualized by DAPI staining. (Scale bar, 10 µm.)

Having observed no change in the VSG switching in populations developmentally compromised through RNAi silencing of the QS signaling pathway, we analyzed parasites more akin to serially passaged monomorphic cell lines used in previous studies (15, 16, 18). Therefore, we exposed the GFP^ESpro^AnTat1.1^ES^ *HYP2*, GFP^ESpro^AnTat1.1^ES^ *NEK*, and GFP^ESpro^AnTat1.1^ES^ *DYRK* RNAi lines to serial passage in vitro. Parasites were maintained for 58 to 73 d by passage, with cell densities maintained initially below 1 × 10^6^ cells/mL but increasing over the course of the passage series to ∼3 × 10^6^ cells/mL (Fig. 5 *A–C*). In each case, blasticidin selection was retained to ensure maintenance of the reporter construct, and doxycycline was excluded to prevent activation of RNAi, which would lead to inducible monomorphism. After the selection period, all 3 populations displayed reduced population doubling times (*SI Appendix*, Fig. S5*A*) and were able to maintain proliferation at higher cell densities compared to the respective parental “start” populations (Fig. 5 *A–C*). Northern blotting confirmed that in each case the increased proliferation of the cells was not accompanied by a loss of stringent RNAi control for the QS genes, with transcript depletion being evident in each population when the cells were induced with doxycycline (*SI Appendix*, Fig. S5*B*). Phenotypic and transcriptomic analysis of the selected populations further confirmed their reduced developmental competence after serial passage. Firstly, the parasites showed progressive resistance to 8pCPT-cAMP–mediated growth arrest over the course of their selection in vitro (a proxy for stumpy formation) (21) (Fig. 5 *D–F* and *SI Appendix*, Fig. S5*C*). Secondly, each cell line exhibited increased virulence in mouse infections as the parasitemias progressed, this being most extreme for the *NEK* reporter line, whereas the *HYP2* and *DYRK* lines exhibited delayed arrest (*SI Appendix*, Fig. S6). Concomitantly, the selected cells remained slender in morphology at high parasitemia (Fig. 5*G*) and expression of the stumpy enriched marker PAD1 protein was reduced or absent for the passaged lines (*SI Appendix*, Fig. S6 *A* and *B*). Finally, RNAseq analysis (*n* = 3 per population) at the start and end of selection by serial passage demonstrated a reduced abundance of transcripts linked to bloodstream developmental competence or differentiation to procyclic forms for the GFP^ESpro^AnTat1.1^ES^ *NEK* line (*SI Appendix*, Fig. S7 and Table S1 and Dataset S1) (22, 23). Overall, these results demonstrated that by serial passage in vitro, monomorphic populations had been directly isolated from isogenic pleomorphic progenitors, these exhibiting reduced developmental capacity in response to the physiological stumpy induction factor signal. We term these lines “selected monomorphs.”

**Fig. 6.**
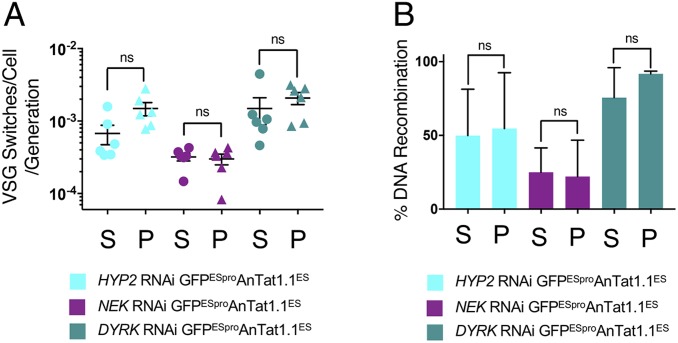
Selected monomorphs do not exhibit reduced VSG switch frequency. (*A*) The calculated VSG switches/cell/generation for the parental and selected GFP^ESpro^AnTat1.1^ES^ *HYP2* (blue), GFP^ESpro^AnTat1.1^ES^ *NEK* (plum), and GFP^ESpro^AnTat1.1^ES^ *DYRK* (teal) RNAi lines. There was no significant difference in VSG switches/cell/generation after selection for monomorphism (*P* > 0.05, Student’s *t* test). “S” represents the “start” pleomorphic population and “P” represents the final “passaged” population (*HYP2*, day 73; *NEK*, day 68; and *DYRK*, day 58). The data represent the mean ± SD (*n* = 6). (*B*) The percentage of switched GFP^ESpro^AnTat1.1^ES^ *HYP2* (blue), *NEK* (plum), and *DYRK* (teal) RNAi cells that had switched their expressed VSG by DNA recombination (i.e., VSG AnTat1.1^−^/GFP^+^). There was no significant difference in the number of VSG switches mediated by DNA recombination following the loss of pleomorphism, *P* > 0.05, Student’s *t* test. ns, not significant, *P* > 0.05.

The selected monomorphic lines were then analyzed for their antigen switch frequency relative to the respective pleomorphic “start” populations. Six replicates for each line were grown in vitro, as were the parental pleomorphic lines from which they were derived by passage. In each case, the number of cell replications in the parental and selected populations were equivalent (pleomorph vs. monomorph replications were: *HYP2*, 25.9 ± 0.3 vs. 25.8 ± 0.8; *NEK*, 26.1 ± 0.7 vs. 25.9 ± 0.6; and *DYRK*, 25.8 ± 0.8 vs. 26.5 ± 0.7). Subsequent analysis of their antigen switch frequency revealed that the GFP^ESpro^AnTat1.1^ES^ *NEK* RNAi selected monomorphs switched at a lower frequency overall than either the GFP^ESpro^AnTat1.1^ES^ *HYP2* or GFP^ESpro^AnTat1.1^ES^ *DYRK* RNAi passaged population, but there was no significant difference (*P* > 0.05, Student’s *t* test) in any population between cells before and after selection (Fig. 6*A*). Thus, although variable VSG switch rates were seen between lines, the de novo selected monomorphs did not change their antigen switch frequency with respect to the isogenic pleomorphic parental populations from which they were derived. Moreover, in the cells that did undergo antigen switching, there was no significant change in the proportion that underwent in situ vs. recombination-based switching (Fig. 6*B*). In conclusion, different cell lines can exhibit different VSG switch frequencies but developmental capacity and antigen switch frequency are independently selected traits.

## Discussion

The major contributors to the infection dynamics of African trypanosomes in their mammalian host are their capacity for immune avoidance by antigenic variation and their generation of quiescent stumpy forms, the prevalence of which influences both parasite virulence and transmissibility. Earlier studies have proposed a link between the frequency of antigenic variation and the laboratory adaptation of bloodstream-form parasites, a phenomenon also seen with *Plasmodium* parasites (24, 25). Laboratory adaptation readily selects for reduced ability to generate stumpy forms (monomorphism) because density-dependent differentiation introduces a fitness cost that is not counterbalanced by the requirement for stumpy forms during parasite transmission through tsetse flies. Although laboratory-adapted lines have formed the basis of most analyses of trypanosome biology and antigenic variation, studies have often relied on the comparison between trypanosome lines derived from independent selections or of unknown passage history. Furthermore, where directly selected monomorphs have been previously analyzed, assays monitoring antigen switch frequency have usually been necessarily indirect and low resolution, being reliant on the antibody-based lysis of unswitched cells then the outgrowth of switched cells in inoculated animals (17). This inevitably creates the potential for over- or under representation of switch events. For the studies reported here, 2 direct comparisons were carried out: using either “inducible monomorphs” or “selected monomorphs” with antigenic variation determined using a direct FACS-based assay. These cells were clonally expanded, avoiding the potential for bottlenecking or the stochastic selection of switched cells. In both cases the comparisons were between isogenic populations, either where RNAi against an identified QS signaling component was induced, or using serially passaged lines directly selected over 50 to 70 d. In both approaches, antigen switch frequency and the developmental capacity were seen to be uncoupled.

Overall, we observed switch frequencies around 10^−3^ to 10^−4^ switches per cell generation. This frequency is higher than often reported in the literature, although different lines and parasites expressing different antigenic variants have been reported to exhibit different switch frequencies even using consistent methodology (15, 26); further, direct comparisons between absolute switching rates in different studies are complicated by differing experimental design and analytical approaches. Regardless, substantial changes in relative switch frequency would be readily detected in our assays as demonstrated by our analysis of the *T. brucei* Lister 427 line engineered to promote antigen switching through I-*Sce*I–mediated ES DNA cleavage. Furthermore, all cells expressed VSG AnTat1.1 at the outset of the switch assay in our assays, minimizing the confounding effects of distinct VSG types exhibiting differential growth rate (26) and apparent switch frequencies.

The 2 parasite-intrinsic components that dominate the trypanosome infection dynamic are the differentiation between slender and stumpy forms, and the rate of antigenic variation (27, 28). Inevitably, the rate of differentiation to stumpy forms influences the overall antigenic switch frequency of the population because stumpy cells are irreversibly committed to cell cycle arrest and cannot generate new variants (28). However, when only proliferative cells are considered, the current data reveal no evidence for mechanistic tethering between developmental capacity and antigen switch frequency. Thus, these components can each contribute significantly to the infection dynamic, but each is independently selected. This introduces the potential for a multivariate normal distribution of switch frequency and relative pleomorphism in different trypanosome isolates in the laboratory and circulating in the field. This may be advantageous for the parasite in different host settings [e.g., host species or host compartments supporting different parasitemias (29) or quorum sensing signal stability (10)], different transmission contexts (e.g., sylvatic or domestic) (30), or in the context of coinfection with competing trypanosomes of the same (9) or different (31) trypanosome species. Uncoupling may also have facilitated the emergence and spread of trypanosomes such as *T. b. evansi*, responsible for *surra*, a disease of camels, horses, buffaloes, and cattle present in Africa, Asia, and South America (i.e., beyond the distribution of tsetse flies). These parasites have lost the ability to undergo cyclical development in the tsetse fly through defects in their mitochondrial genome and instead rely on transmission via the blood-contaminated mouthparts of biting flies such as tabanids (32). These nontsetse-transmitted parasites exhibit monomorphism (33), their reduced ability to generate stumpy forms contributing to the higher parasitemias that support mechanical transmission. Nonetheless, although some hosts display acute infection profiles, others exhibit chronic infections, including livestock and buffalo hosts, for example. For such parasites, a sustained high level of antigen switching combined with the elevated parasitemias characteristic of parasites with reduced developmental competence would provide a strong selective advantage supporting both long-term immune evasion and effective disease spread by nontsetse biting flies.

## Materials and Methods

### Trypanosomes.

The pleomorphic *T. brucei* AnTat1.1 (EATRO1125) 90:13 trypanosome strain (34) was used for generating “inducibly monomorphic” and selected monomorphic cell lines in this study. *T. brucei* Lister 427 monomorphic trypanosomes were acquired from Achim Schnaufer, University of Edinburgh.

Bloodstream-form trypanosome cultures were maintained in HMI-11 medium (35) supplemented with 20% FCS (Gibco), 100 U/mL penicillin and streptomycin, and the appropriate selective drug(s) following transfection. Overall, 2 to 5 × 10^7^ cells were used for each transfection, with cells being resuspended in 50 μL of Amaxa transfection buffer (Amaxa Basic Parasite Nucleofector Kit 2) and 10 μL of the linearized DNA, and then electroporated in an Amaxa Nucleofector II using the Z-001 program. *T. brucei* EATRO 1125 AnTat1.1 90:13 cells were maintained in 0.5 µg/mL hygromycin, 2.5 µg/mL G418, 0.5 µg/mL puromycin, and 10 µg/mL blasticidin. Lister 427 VSGup pLF12 eGFP_BLA cells were maintained in 20 µg/mL phleomycin, 2.5 µg/mL hygromycin, 2 µg/mL puromycin, and 10 µg/mL blasticidin. Pleomorphic strains were maintained below densities of 1 × 10^6^ cells/mL and monomorphic strains below densities of 2 × 10^6^ cells/mL

All infections were performed according to the UK Home Office and Project License (PPL number 604373) regulations in accordance with the local ethical approval requirements of the University of Edinburgh. Blood stocks were prepared after inoculation of mice with 10,000 parasites. Depending on the virulence of lines, experimental infections were initiated with 12,500 to 750,000 parasites in a total volume of 200 µL. Infections were monitored daily by the rapid matching method of Herbert and Lumsden (36).

### In Vitro 8pCPT-cAMP Resistance Assays.

Assays were performed as described in Mony et al. (11)

### Flow Cytometry and VSG Switch Assays.

A total of 2 to 5 × 10^6^ culture-derived cells were centrifuged at 1,000 × *g* and washed 3 times in 1× PBS. After washing, cells were resuspended in 500 μL 2% formaldehyde/0.05% glutaraldehyde in 1× PBS overnight at 4 °C in the dark. After fixation, the cells were washed twice with 1× PBS and then blocked at room temperature for 30 min in 2% BSA/1× PBS. For detection of VSG AnTat1.1 expression, the cells were resuspended in 200 μL of 1:20,000 diluted α-VSG AnTat1.1 in 2% BSA/1× PBS and incubated at 4 °C for 30 min, washed twice more with 1× PBS, and then incubated with 200 μL of 1:1,000 diluted α-rabbit secondary antibody conjugated to Cy5 (Jackson ImmunoResearch) in 2% BSA/1× PBS for a further 30 min. The α-VSG 221 antibody was conjugated to the Alexa Fluor 647 fluorophore. After the block and wash step, the cells were incubated in 200 μL of 1:5,000 diluted α-VSG 221/Alexa Fluor 647 for 30 min in the dark. After incubation with the respective fluorophores, the cells were washed 3 times with 1× PBS and resuspended in 500 μL of filtered 1× PBS. Samples were processed on an LSRII Flow Cytometer (BD Biosciences) and 10,000 events acquired. Analysis was performed using FlowJo 10.4.2 software. Data were filtered using the forward (FSC) and side scatter (SSC) profiles to ensure that only intact and singlet cells were analyzed, with the gates for VSG AnTat1.1, VSG 221, or GFP determined based on the control samples. To assay VSG switch frequency, 2 × 10^7^ cells were processed as before, except that the blocking and antibody steps were performed in volumes of 1 mL. The labeled cells were resuspended in 1 mL of filtered 1× PBS, and to each experimental tube, 40 μL (∼5 × 10^4^) of vortexed CountBright Absolute Counting Beads (Thermo Fisher Scientific) was added. At least 1,000 bead events were collected per experimental sample. Samples were processed on an LSRII Flow Cytometer (BD Biosciences) and 1,000,000 events were acquired for each experimental sample and the positive control. A total of 10,000 events were collected for the negative and secondary-only controls. The calculations used to extrapolate the number of VSG switches/cell from the gated FACS data were based on the method described in ref. 37. Briefly, the absolute number of VSG AnTat1.1 negative cells in the population (as calculated by ((number of beads added × number of VSG AnTat1.1 negative cells acquired)/number of beads acquired) × dilution factor) was divided by the absolute number of cells in the population (as calculated by ((number of beads added × number of cells acquired)/number of beads acquired) × dilution factor), where “dilution factor” refers to the proportion of the final VSG switch assay culture that was removed and fixed for FACS analysis. To convert this value to VSG switches/cell/generation, the number of VSG switches per cell was divided by the number of population doublings that occurred during the VSG switch assay growth phase.

### Northern Blotting.

*HYP2*, *NEK*, and *DYRK* Northern blotting was performed as described in McDonald et al. (38).

### Microscopy Analysis.

Methanol-fixed blood smears were removed from methanol, rehydrated in 1× PBS for at least 5 min, and then stained with ∼50 µL 10 µg/mL 4′,6-diamidino-2-phenylindole (DAPI) in 1× PBS for 2 min in the dark. The slides were washed once in 1× PBS and mounted with Mowiol containing DABCO. The slides were dried overnight in the dark and then stored at 4 °C until use.

Cells were visualized under a Zeiss Imager Z2 microscope and 250 cells counted per sample. Images were captured using a QImaging Retiga 2000R camera and edited with ImageJ software (NIH).

### Western Blotting.

PAD1 Western blotting was performed as described in McDonald et al. (38).

### RNAseq and VSGseq.

For the RNAseq analysis, RNA was prepared from triplicate populations of the parental “start” GFP^ESpro^AnTat1.1^ES^ *NEK* RNAi cells, the monomorphic “end” GFP^ESpro^AnTat1.1^ES^ *NEK* RNAi cells using the Qiagen RNeasyMini Kit as per the manufacturer’s instructions. The isolated RNA was TURBODNase treated according to the manufacturer’s protocol and RNAseq was performed by BGI Tech Solutions (Hong Kong) using the “Eukaryotic Transcriptome Resequencing HiSEq. 4000 PE100” service. Quality control of the raw data was performed using the Fast QC program (Babraham Bioinformatics), and paired end reads were trimmed using cutadapt (39). Reads were aligned to the *T. b. brucei* TREU927 genome using Bowtie 2 (40) to obtain counts for the number of reads that mapped to each gene. These counts were normalized to the reads per kilobase of transcript per million mapped reads (RPKM) to account for gene size and read depth (Dataset S1). The data discussed in this manuscript have been deposited in NCBIs Gene Expression Omnibus and are accessible through GEO series accession number GSE134892 (https://www.ncbi.nlm.nih.gov/geo/query/acc.cgi?acc=GSE134892).

Processing of the VSGseq data was through the pipeline provided at https://github.com/mugnierlab/VSGSeqPipeline/blob/master/VSGSeqPipeline.py (41). Adapters (from the tagmentation step) and SP6 sequences (from the VSG PCR step) were trimmed using trim_galore (Babraham Bioinformatics) and cutadapt (39), respectively. Sequencing reads were assembled by Trinity (42) and ORFs (defined as a start codon to stop codon or a start codon to the end of a contig) of >900 bp were identified. These ORFs were compared against the *T. b. brucei* EATRO1125_vsgs reference database (http://129.85.245.250/Downloads/vsgs_tb1125_all_atleast150aas_cds.txt) generated using available *T. brucei* EATRO1125 sequences deposited in GenBank (https://www.ncbi.nlm.nih.gov/genbank/) using BLASTn to identify VSG sequences. Sequences that matched chromosomal sequences or non-VSG sequences were removed. VSGs with >98% sequence identity were merged using cd-hitest (43). These final merged contigs represented an individual VSG, which was included in the reference genome. The reads for each sample were aligned to the reference genome using Bowtie, allowing for only uniquely mapping reads and no more than 2 mismatches per read. Quantitation exploited MULTo (44) which corrects for the mappability of each VSG and was used to determine RPKM values. The output MULTo-analyzed csv file showed the expression of each VSG in each sample, both in terms of RPKM and percentage of the population (RPKM for that VSG/total RPKM), and the BLAST similarity results for each de novo-assembled VSG compared to the most similar reference VSG.

### Quantitative RT-PCR.

The SuperScript III First-Strand Synthesis System (Invitrogen) and an oligo(dT)_20_ primer were used for first-strand cDNA synthesis according to the manufacturer’s instructions. To remove cRNA, the single-stranded cDNA was incubated with 2U of *Escherichia coli* RNase H at 37 °C for 20 min.

Quantitative RT-PCR reactions were performed on a LightCycler 96 machine (Roche) using the Power SYBR Green PCR Master Mix (Applied Biosystems) and gene-specific primers. cDNA was used at a concentration of 1:20. Transcript abundances were quantified relative to Tb*ZFP3* using the ΔΔCT method.

## Supplementary Material

Supplementary File
