# Supplementary material for: Developmental competence and antigen switch frequency can be uncoupled in Trypanosoma brucei
Source: Proc Natl Acad Sci U S A. 2019 Oct 21;116(45):22774–82. doi: 10.1073/pnas.1912711116 (PMC6842576; doi:10.1073/pnas.1912711116)
Supplement: Supplementary File [file pnas.1912711116.sapp.pdf]

PNAS
www.pnas.org

Supplementary Information for

Developmental competence and antigen switch frequency can be uncoupled in *Trypanosoma brucei*

Kirsty R. McWilliam, Alasdair Ivens, Liam J. Morrison, Monica R. Mugnier and Keith R. Matthews

*Keith R. Matthews; keith.matthews@ed.ac.uk;
Telephone: +441316513639;
ORCID 0000-0003-0309-9184

*Monica Mugnier; mmugnie1@jhu.edu
Telephone: +1-410-614-4894
ORCID 0000-0002-1535-2530

This PDF file includes:

Figures S1 to S7
Tables S1
Legends for Dataset S1

Other supplementary materials for this manuscript include the following:

Datasets S1

www.pnas.org/cgi/doi/10.1073/pnas.1912711116

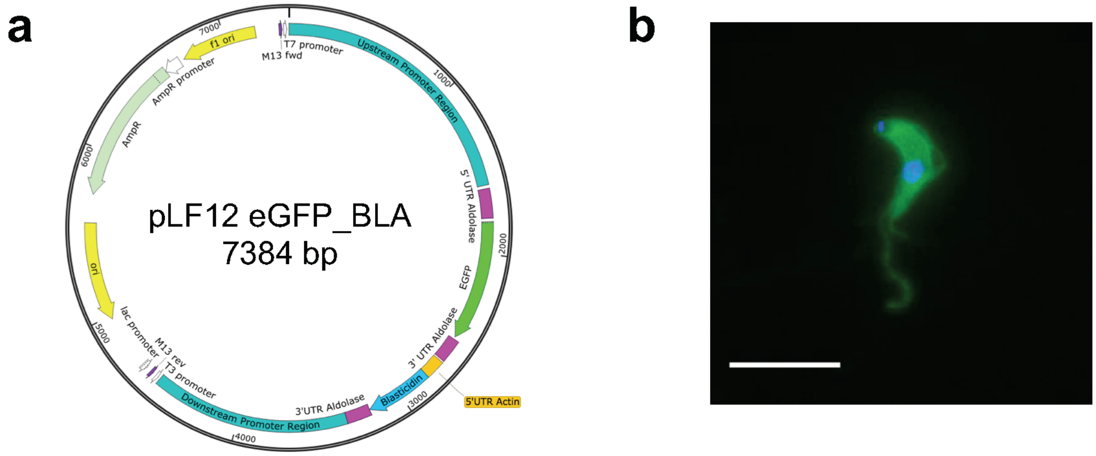

Fig. S1. **GFP^{ESpro}AnTat1.1^{ES} H*YP2*, *NEK* and *DYRK* RNAi cell lines express GFP from the VSG AnTat1.1 ES**

a. pLF12 eGFP_*BLA* encodes eGFP and blasticidin resistance.

b. When the VSG AnTat1.1 ES was active, the cells emitted a strong cytoplasmic GFP signal. Bar, 10µm.

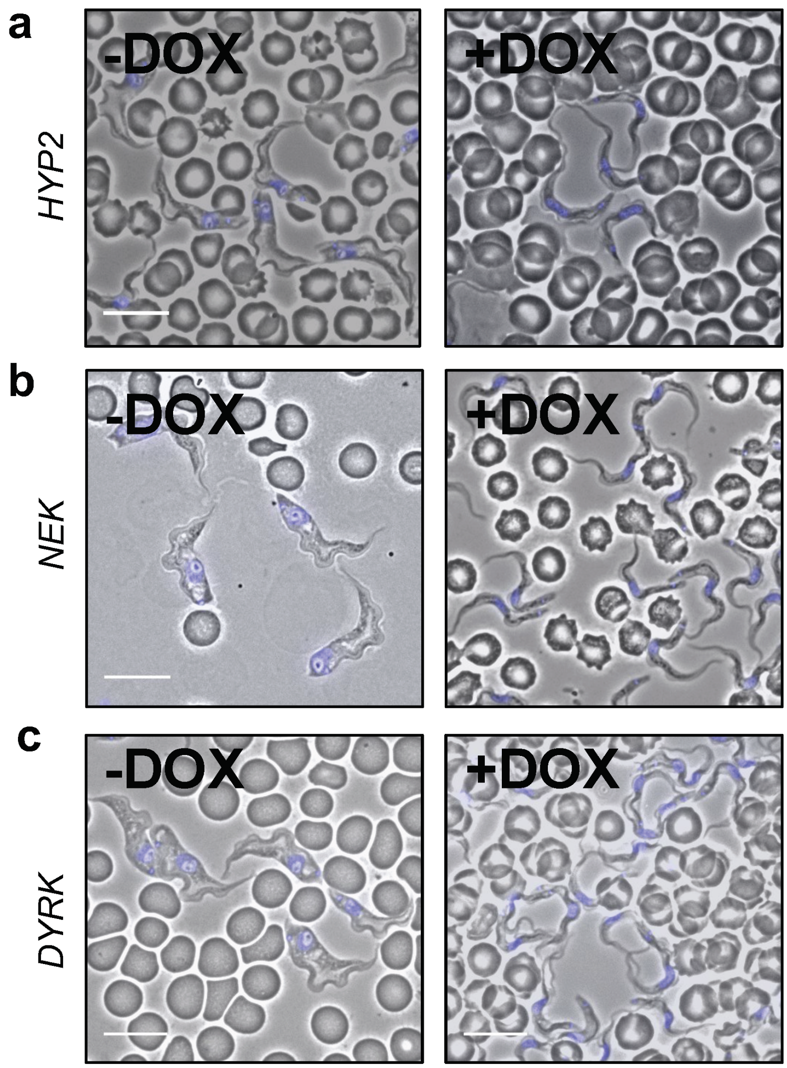

Fig. S2. **The GFP^{ESpro}AnTat1.1^{ES} *HYP2*, *NEK* and *DYRK* RNAi lines are inducibly monomorphic**

a-c. Final day cell morphology of the GFP^{ESpro}AnTat1.1^{ES} *HYP2* (a), GFP^{ESpro}AnTat1.1^{ES} *NEK* (b) and GFP^{ESpro}AnTat1.1^{ES} *DYRK* (c) RNAi cell lines. The mice were sacrificed before the parasitaemia became lethal and the trypanosomes were purified from the blood on day 5 (*DYRK* RNAi) or day 6 (*HYP2* and *NEK* RNAi). Induction of RNAi generated populations of cells which grew to high parasitaemia and were slender in morphology. Uninduced populations of cells restricted their parasitaemia from day 4 onwards and were enriched for morphologically stumpy forms. Bar, 10μm

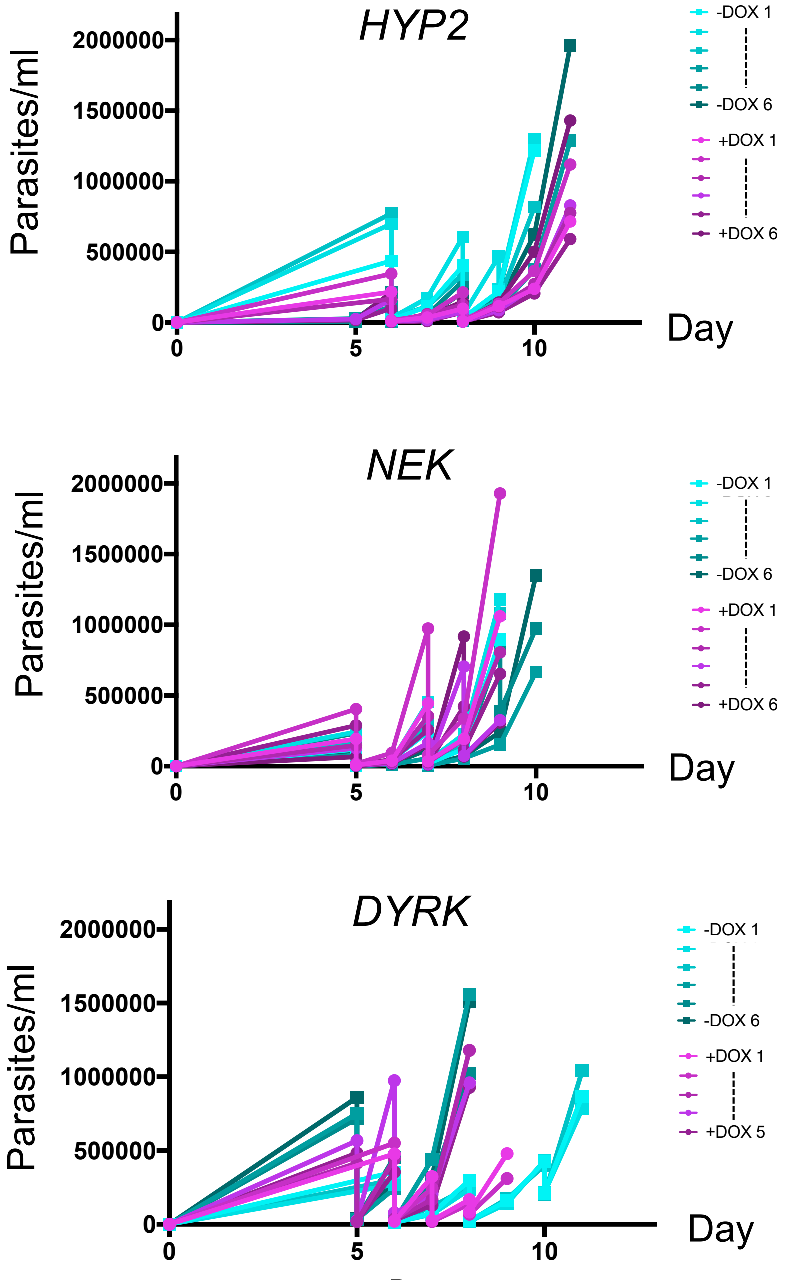

Fig. S3. **The induction of monomorphism did not significantly reduce VSG switch rate nor change the predominant mechanism of VSG switching**

VSG switch assay growth of the GFP^{ESpro}AnTat1.1^{ES} *HYP2*, GFP^{ESpro}AnTat1.1^{ES} *NEK* and GFP^{ESpro}AnTat1.1^{ES} *DYRK* RNAi lines. Uninduced populations are shaded in teal and induced in plum. Assays were performed twice in triplicate (n=6), except for the induced *DYRK* RNAi experiment where one assay was performed in triplicate and one in duplicate (n=5). Additional culture media was added periodically to maintain cell growth- the cultures were not subdivided.

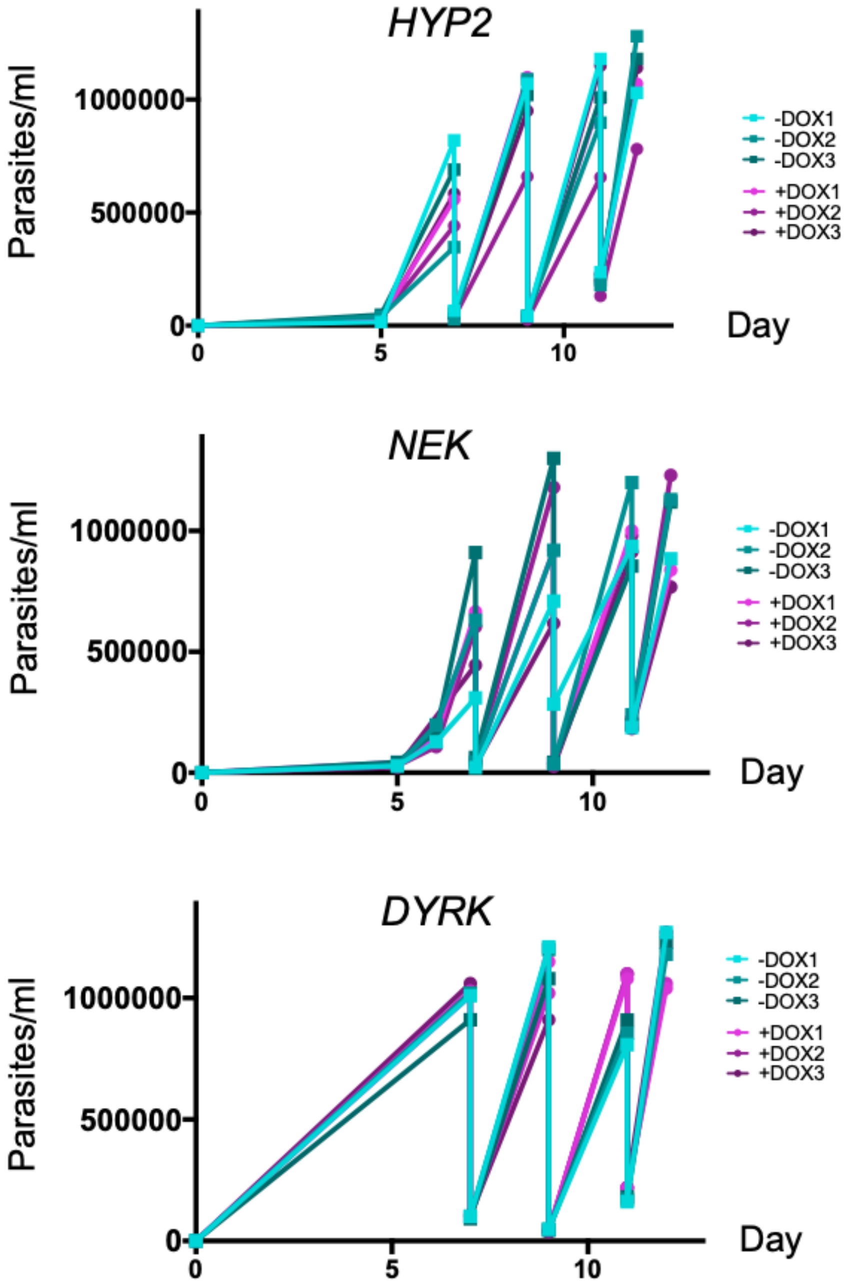

Fig. S4. **The induction of monomorphism did not generate a change in the frequency or diversity of expressed VSG in the populations**

In vitro growth of the $GFP^{ESpro}AnTat1.1^{ES}$ *HYP2*, $GFP^{ESpro}AnTat1.1^{ES}$ *NEK* and $GFP^{ESpro}AnTat1.1^{ES}$ *DYRK* RNAi lines (± induction) prior to pre-MACS RNA extraction. Uninduced populations are shaded in teal and induced in plum. Assays were performed in triplicate. Additional culture media was added periodically to maintain cell growth- the cultures were not subdivided.

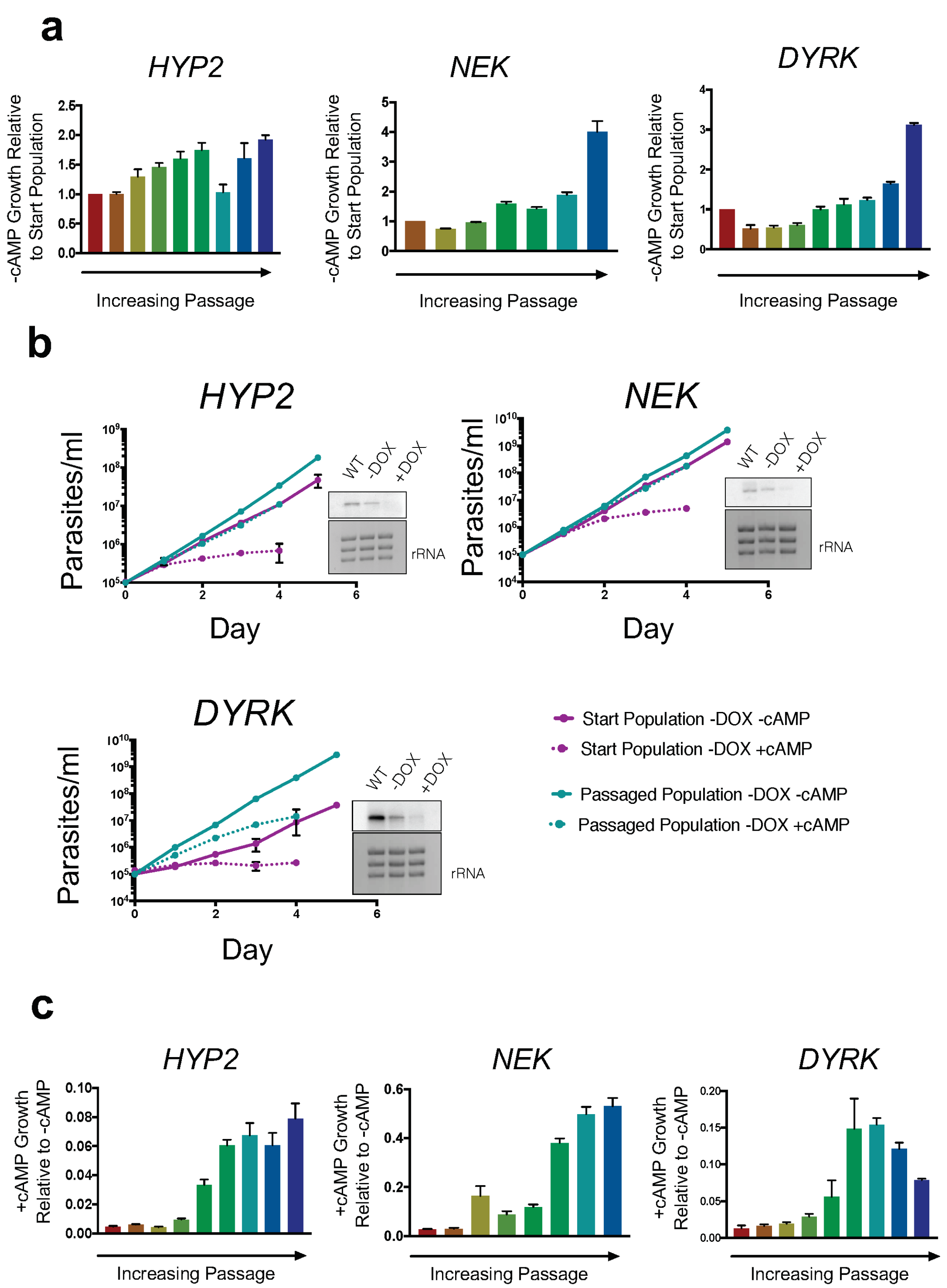

Fig. S5. **Long term *in vitro* passage selects for cells with reduced 8-pCPTcAMP sensitivity and increased growth rate.**

a. Relative growth of the intermediate (isolated at 1 week intervals during selection) and final selected populations compared to the parental population. With increasing passage number, the generation time decreased. The data represent the mean ± S.D.

(n=3). The *NEK* dataset is missing the start and +1 week population data and therefore the values were normalised to the +2 week growth values.

b. 8-pCPTcAMP response cumulative growth curves of pleomorphic start and selected $GFP^{ESpro}AnTat1.1^{ES}$ populations. Treatment with 8-pCPTcAMP caused the pleomorphic parental 'start' populations to arrest. Passaged populations showed varying degrees of resistance to the compound. The 8-pCPTcAMP was added on day 1. The gene targeted by the RNAi machinery is denoted above the respective graph. Data represent the mean±S.D (n=3). Inset Northern blot detection of *HYP2*, *NEK* and *DYRK* transcripts in the selected populations. A pleomorphic *T. brucei* AnTat1.1 90:13 line was used as the WT control. The RNAi machinery remained inducible and therefore knock down of a stumpy formation gene could not explain the acquired 8-pCPTcAMP resistance. rRNA was used as a loading control.

c. Relative growth of the start and intermediate populations (isolated at 1 week intervals during selection) in the presence of 8-pCPTcAMP compared to the growth of the respective populations in the absence of the compound. With increasing passage, resistance to 8-pCPTcAMP increased. The data represent the mean ± S.D. (n=3). The *HYP2* dataset is missing the pleomorphic 'Start' population and therefore values were normalised to the + 1 week population. The *NEK* dataset is missing the start and +1 week population data and therefore the values were normalised to the +2 week growth values.

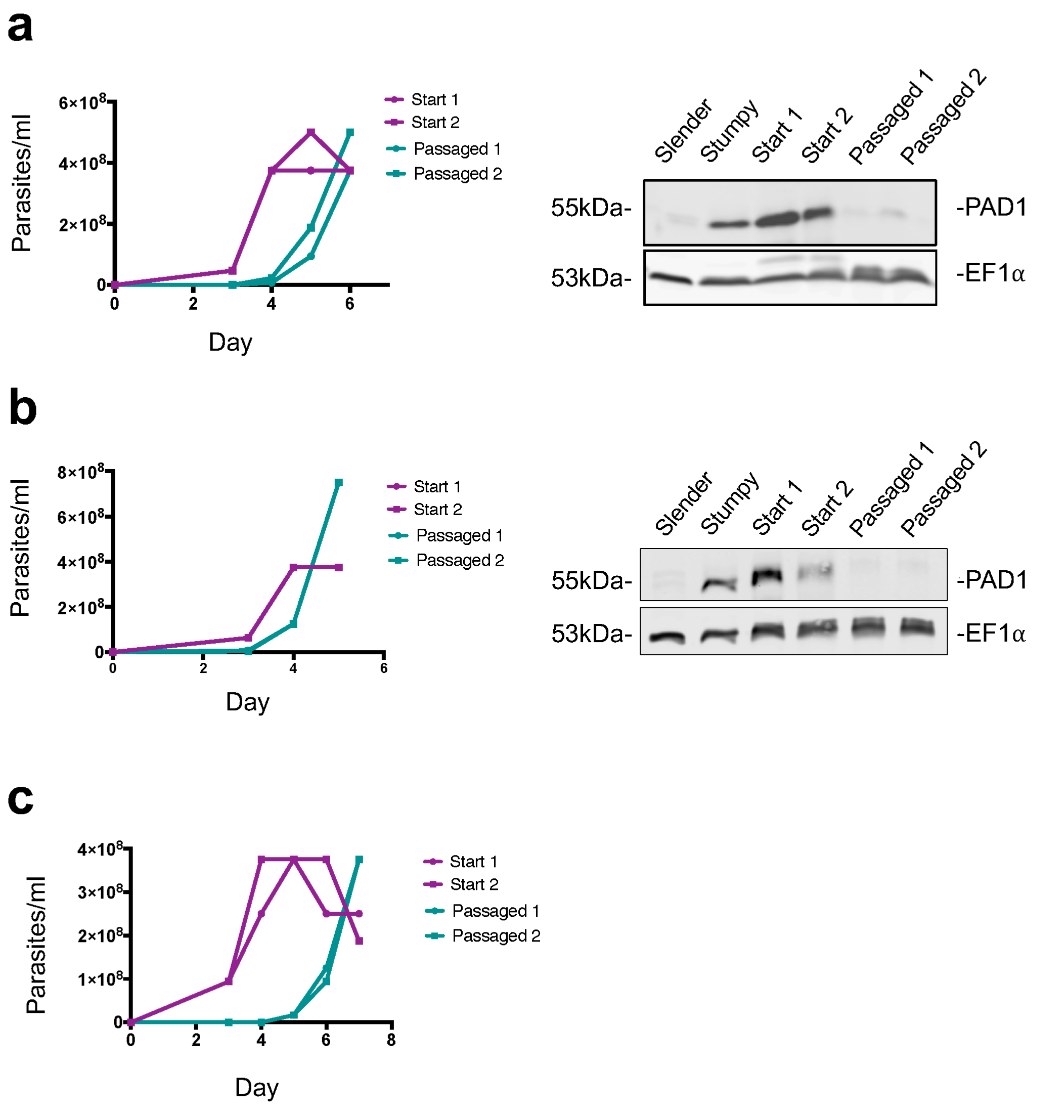

Fig. S6. **GFP^{ESpro}AnTat1.1^{ES} *HYP2, NEK and DYRK* RNAi populations have reduced sensitivity to SIF *in vivo*.**

a. (Left) *In vivo* growth of the GFP^{ESpro}AnTat1.1^{ES} *HYP2* RNAi selected population compared to the pleomorphic parental cells. The selected population's parasitaemia continued to ascend over the infection time course; less cells were inoculated to initiate infection so that pleomorphic and monomorphic cells could be harvested for comparison on day 6 of infection. The pleomorphic parental cells arrested in response to SIF. (Right) Western blot detection of PAD1. The passaged population did not express PAD1 on its surface on the final day of infection. Slender and stumpy *T. brucei* AnTat1.1 90:13 WT cells served as controls. Detection of EF1α was performed as a loading control.

b. As panel a, but for the GFP^{ESpro}AnTat1.1^{ES} *NEK* RNAi line

c. *In vivo* growth as panel a, but for the GFP^{ESpro}AnTat1.1^{ES} *DYRK* RNAi line. Western blot detection for PAD1 was not performed for the passaged GFP^{ESpro}AnTat1.1^{ES} *DYRK* RNAi line.

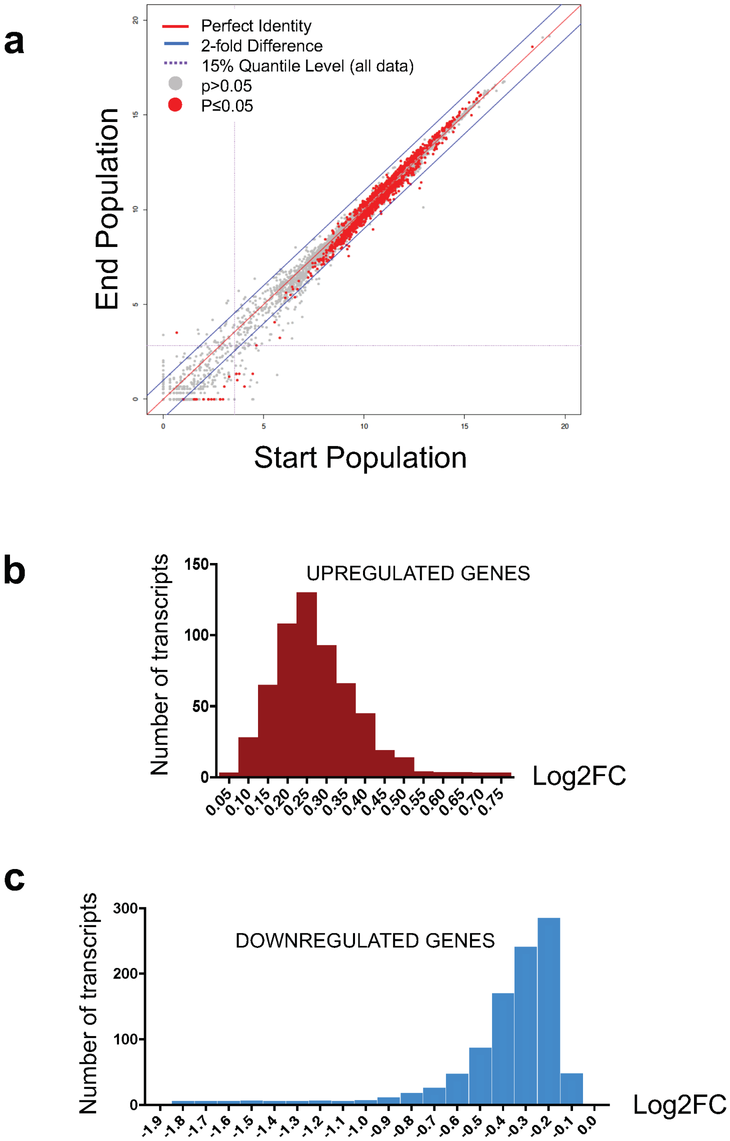

Fig. S7. **GFP^{ESpro}AnTat1.1^{ES} *NEK* RNAi selected monomorph gene expression relative to the pleomorphic parental population.**

a. Each transcript is denoted by a dot, grey when the p-value was >0.05 and red when the p-value was ≤0.05. The red horizontal line shows where gene expression was identical between the two populations and the two blue lines represent two-fold increases or decrease in expression. Before pseudogenes and VSG genes were removed from the analysis, 950 genes were recognised as being downregulated (16 over 2-fold) and 578 upregulated (over 2-fold).

b. Transcripts upregulated in the selected monomorphs compared to the parental pleomorphs (pseudogenes and VSG genes were removed from the list).

c. Transcripts downregulated in the selected monomorphs compared to the parental pleomorphs (pseudogenes and VSG gene were removed from the list).

Table S1. Annotated transcripts downregulated in the GFP^{ESpro}AnTat1.1^{ES} *NEK* RNAi selected monomorphs that are linked to bloodstream developmental competence or differentiation to procyclic forms.

GENE CODE	ANNOTATION	LOG2 FC	ADJUSTED P VALUE	GENE FUNCTION
Tb927.6.510	GPEET procyclin	-1.63	0.0003	Major GPI-anchored surface coat protein in procyclic forms (1).
Tb927.9.7470	TbNT10	-1.06	0.0003	Purine nucleoside transporter (2).
Tb927.7.5940	Protein Associated with Differentiation 2 (PAD2)	-0.97	0.0003	Recieves CCA procyclic differentiation signal(3).
Tb927.10.10260	EP1 procyclin	-0.96	0.0056	Major GPI-anchored surface coat protein in procyclic forms (1)
Tb927.9.11050	4E-interacting protein (4EIP)	-0.78	0.0017	Contributes to translation repression during differentiation to stumpy form(4).
Tb927.7.5930	Protein Associated with Differentiation 1 (PAD1)	-0.67	0.0014	Expressed on surface of stumpy forms (3)
Tb927.5.320	Receptor-type adenylate cyclase (GRESAG 4)	-0.64	0.0024	Midgut specific adenyl cyclase. Marker for late procyclic stage (5)
Tb927.11.2690	Succinyl-CoA:3-ketoacid coenzyme A transferase	-0.64	0.0229	Mitochondrial enzyme (6)
Tb927.7.6830	Trans-sialidase (putative)	-0.64	0.0006	Membrane-bound and expressed on surface. Enable procylic cells to utilise host sialic acid (7)
Tb927.9.5900	Glutamate dehydrogenase	-0.63	0.0029	Mitochondrial enzyme(8).

Dataset S1. RNA-seq data for GFP^{ESpro}AnTat1.1^{ES} *NEK* RNAi cells before or after selection in culture. The data have been deposited in NCBIs Gene Expression Omnibus and are accessible through GEO series accession number GSE134892 (https://www.ncbi.nlm.nih.gov/geo/query/acc.cgi?acc=GSE134892).

References

1. Butikofer P, Ruepp S, Boschung M, & Roditi I (1997) 'GPEET' procyclin is the major surface protein of procyclic culture forms of Trypanosoma brucei brucei strain 427. *Biochem J* 326 (Pt 2):415-423.
2. Spoerri I*, et al.* (2007) Role of the stage-regulated nucleoside transporter TbNT10 in differentiation and adenosine uptake in Trypanosoma brucei. *Mol Biochem Parasitol* 154(1):110-114.
3. Dean S, Marchetti R, Kirk K, & Matthews KR (2009) A surface transporter family conveys the trypanosome differentiation signal. *Nature* 459(7244):213-217.
4. Terrao M*, et al.* (2018) The suppressive cap-binding complex factor 4EIP is required for normal differentiation. *Nucleic Acids Res* 46(17):8993-9010.
5. Imhof S, Knusel S, Gunasekera K, Vu XL, & Roditi I (2014) Social motility of African trypanosomes is a property of a distinct life-cycle stage that occurs early in tsetse fly transmission. *PLoS Pathog* 10(10):e1004493.
6. Vertommen D*, et al.* (2008) Differential expression of glycosomal and mitochondrial proteins in the two major life-cycle stages of Trypanosoma brucei. *Mol Biochem Parasitol* 158(2):189-201.
7. Engstler M, Reuter G, & Schauer R (1993) The developmentally regulated trans-sialidase from Trypanosoma brucei sialylates the procyclic acidic repetitive protein. *Mol Biochem Parasitol* 61(1):1-13.
8. Mantilla BS*, et al.* (2017) Proline Metabolism is Essential for Trypanosoma brucei brucei Survival in the Tsetse Vector. *PLoS Pathog* 13(1):e1006158.
